# Somatostatin and Somatostatin Receptors: From Signaling to Clinical Applications in Neuroendocrine Neoplasms

**DOI:** 10.3390/biomedicines9121810

**Published:** 2021-12-01

**Authors:** Maria Isabel del Olmo-Garcia, Stefan Prado-Wohlwend, Alexia Andres, Jose M. Soriano, Pilar Bello, Juan Francisco Merino-Torres

**Affiliations:** 1 Endocrinology and Nutrition Department, University and Politecnic Hospital La Fe (Valencia), 46026 Valencia, Spain; alexia.andres@gmail.com (A.A.); merino_jfr@gva.es (J.F.M.-T.); 2 Joint Research Unit on Endocrinology, Nutrition and Clinical Dietetics, University of Valencia-Health Research Institute La Fe, 46026 Valencia, Spain; jose.soriano@uv.es; 3 Nuclear Medicine Department, University and Politecnic Hospital La Fe (Valencia), 46026 Valencia, Spain; praduso@hotmail.es (S.P.-W.); bello_pil@gva.es (P.B.); 4 Food & Health Lab, Institute of Materials Science, University of Valencia, 46980 Valencia, Spain

**Keywords:** neuroendocrine neoplasms, somatostatin receptors, peptide receptor radionuclide therapy, somatostatin analogues, somatostatin antagonists, 68Ga PET, LU-DOTA-TATE

## Abstract

Neuroendocrine neoplasms (NENs) are heterogeneous neoplasms which arise from neuroendocrine cells that are distributed widely throughout the body. Although heterogenous, many of them share their ability to overexpress somatostatin receptors (SSTR) on their cell surface. Due to this, SSTR and somatostatin have been a large subject of interest in the discovery of potential biomarkers and treatment options for the disease. The aim of this review is to describe the molecular characteristics of somatostatin and somatostatin receptors and its application in diagnosis and therapy on patients with NENs as well as the use in the near future of somatostatin antagonists.

## 1. Introduction

Neuroendocrine neoplasms (NENs) are heterogeneous neoplasms which arise from neuroendocrine cells that are distributed widely throughout the body. They have been usually described as infrequent tumors but their incidence has been rising over time up to around 3.65 per 100,000 individuals per year [1,2]. Accordingly, the World Health Organization (WHO) in 2017 and 2019, classified pancreatic and gastrointestinal NENs respectively as well differentiated neuroendocrine tumors (NETs) subclassified as G1, G2 and G3 (according proliferative index and mitotic rate) and poorly differentiated neuroendocrine carcinomas (NECs) [3]. Although heterogenous, many of them share their ability to overexpress somatostatin receptors (SSTR) on their cell surface. Five Somatostatin receptors have been identified: SSTR1, SSTR2, SSTR3, SSTR4 and SSTR5. Somatostatin (SST) is a regulatory peptide that plays a key role in maintaining homeostasis in the body. It keeps the neuroendocrine system in balance, aids in neurotransmission and memory formation, and keeps cellular proliferation in check. Through interaction with SSTR on target cells, it inhibits the release of other peptides and activates pathways of cell cycle arrest. After SST binds to SSTR, a cascade of downstream effects inside the cell takes place in which various enzymes and other proteins participate [4].

Due to the overexpression of SSTR in NENs, this small peptide and its receptor have been a large subject of interest in the discovery of potential biomarkers and treatment options for the disease. SSTR2 and SSTR5 have become biological targets for pharmacological treatment of NENs since the earlies 1980s with somatostatin analogues (SSAs). Furthermore, SSTR2 is a prototypical for the development of peptide radionuclides, used not only for diagnostic purposes, but also for therapeutic interventions, which is the cornerstone of the concept of peptide receptor radionuclide therapy (PRRT). Future investigations are directed to investigate the use of somatostatin antagonists both for diagnostic imaging and treatment in these patients.

The aim of this article is to describe the molecular characteristics of somatostatin and somatostatin receptors and its application in diagnosis and therapy on patients with NENs.

## 2. Somatostatin and Somatostatin Receptors

**Figure 1:**
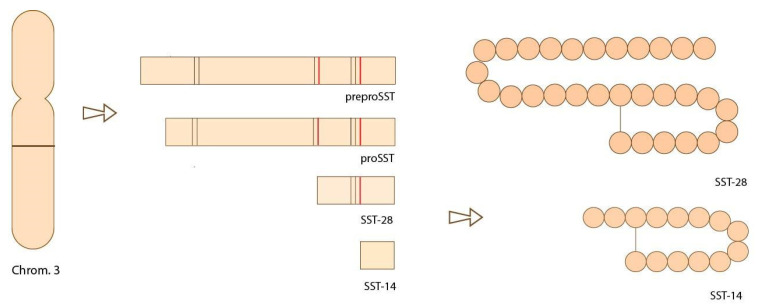
Representation of the location of the gene that codes for SST on chromosome three. DNA is transcribed into RNA and then translated to produce the peptide “pre-proSST”. It is cleaved enzymatically at locations indicated in red, producing isoforms SST-14 and SST-28 [5].

In humans, the gene that codes for the production of somatostatin (SST) is located on chromosome 3q27.3 in humans [5]. This gene holds the instructions for the production of the initial precursor protein called preprosomatostatin (preproSST) through a process of transcription of the genetic code and translation into amino acids, which are the building blocks of the peptide. This peptide is then cleaved at specific sites (Figure 1) to create the prohormone form known as prosomatostatin (proSST). It is further cleaved enzymatically to produce the two bioactive forms; SST-14 and SST-28, characterized by chains of 14 and 28 amino acids, respectively [6]. The functions of both isoforms are similar, with some exceptions in their function in different parts of the body. For example, SST-14 has been found to function predominantly in the brain, while SST-28 executes its function primarily in the gastrointestinal tract [7].

SST is produced in various regions of the body, including the hypothalamus in the brain, the pancreas, throughout the gastrointestinal (GI) tract, other regions of the central and peripheral nervous systems (CNS, PNS) and in smaller quantities in other organs, including the kidney [8]. Due to its vast distribution, it carries out many functions in the body, exhibiting inhibitory effects in various organs. In the pancreas, SST inhibits the release of glucagon and insulin, hormones that are important for the regulation of blood sugar levels and energy metabolism. In the stomach, it inhibits gastrin and histamine, which diminishes acid production, in order to halt the digestive process. In other parts of the digestive tract, it inhibits release of ghrelin, cholecystokinin (CCK), glucose-dependent insulin polypeptide (GIP), vasoactive intestinal peptide (VIP) and pepsin, all of which have functions in the process of digestion and hunger regulation. In the brain, it inhibits neuronal firing as well as the release of growth hormone and prolactin, which play a role in cellular proliferation and growth [6,7,8,9]. In this way, it acts to regulate homeostasis all around the body.

**Figure 2:**
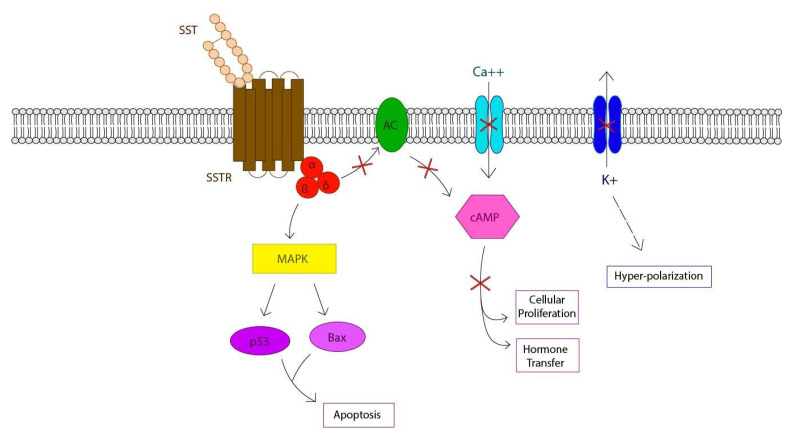
Representation of the ligand–receptor interaction between SST and SSTR (lock and key), which sets off a cascade of signals inside the cell. Inhibition of AC activity and Ca2+ influx results in cell cycle arrest and halts hormone transfer as indicated. Accumulation of K+ inside the cell causes hyper-polarization and pauses communicative activity. Apoptosis, or cell death, is favored. X = cascade signaling which is blocked when SST interacts with SSTR.

When SST is produced, it is secreted out of its cell of origin into the bloodstream through capillaries, which are small blood vessels that exchange blood and compounds to and from tissues. Once in the bloodstream, it can travel throughout the body and target cells in specific organs. These so-called “target cells” are characterized by SST receptors (SSTRs) embedded in their membrane [10]. SSTRs are a class of G protein-coupled receptors consisting of seven α-helical transmembrane domains, with ~20–25 amino acids, starting outside the cell and finishing on the inside (Figure 2). The peptide fits in like a lock and key and sets off a cascade of downstream signals inside the cell cytoplasm [9,10].

In humans, there exist five subtypes of SSTR; SSTR1 through SSTR5, with gene location as are 14q13, 17q24, 22q13.1, 20p11.2 and 16p13.3, respectively. These receptors are primarily found along the GI tract, in the brain, adrenals, and the pancreas. Each of these receptors has a strong binding affinity for both SST isoforms, but specifically SSTR1–4 and SSTR5 have higher selectivity for SST-14 and SST-28, respectively [9,10].

Inside the cell, attached to the receptor, is a heterotrimer which consists of α, β and γ subunits which dissociates from the receptor when activated by the ligand. This activity signals for a cascade of downstream effects inside the cell in which various enzymes and other proteins respond. For example, interaction of SST with its G-protein coupled receptor leads to inhibition of intracellular Ca2+ influx and adenylate cyclase (AC) activity inside the cell, which regulate cyclic AMP (cAMP) levels. cAMP is involved in intracellular signaling such as the transfer of hormones inside cells and cellular proliferation [6]. It can also inhibit K+ efflux, leading to hyper-polarization of the membrane, which inhibits the action potential, making it unable to send signals (in the case of neurons). These processes are balanced by activating signals from other peptides in order to maintain equilibrium inside the cell and the rest of the body.

SST has also been found to inhibit cellular proliferation and alter growth factor signaling through the regulation of enzymes like activation of protein tyrosine phosphatase (PTPs) and the mitogen-activated protein kinase (MAPK) pathway. Interaction with these and other proteins such as tumor protein (p53) and Bcl-2 associated X protein (Bax) leads cells to undergo apoptosis or cell death [6]. In one study, SST was found to inhibit adhesion of cancerous cells to blood vessels, inhibiting the metastasis of certain tumors. The anti-proliferative effects on cells have made it a subject of increased attention for clinical practices and potential future applications [11].

These properties make somatostatin a potential candidate for diagnostic and therapeutic purposes in various diseases. Currently, it is used to treat Cushing’s disease, acromegaly, upper gastrointestinal bleeding, and neuroendocrine neoplasms (NENs).

## 3. Somatostatin Receptors as Targets for Imaging Diagnosis on Neuroendocrine Neoplasms

NENs are heterogeneous and complex malignancies; however, many of them share their ability to overexpress somatostatin receptors (SSTR) on their cell surface [11]; this provides a unique and specific molecular target for imaging. Nuclear medicine is based on the use of radiotracers or radiopharmaceuticals, which are formed by the union of a drug and a radioactive isotope, which after being injected into the patient, allow the acquisition of molecular or functional images.

Octreotide, a somatostatin analogue, was first radiolabelled in 1983 allowing SSTR imaging with a nuclear medicine gamma camera [12]. 111In-labeled somatostatin analogues were firstly developed: the best known is the 111In-labeled octreotide and diethylenetriaminepentaacetic (DTPA) chelator, called 111In-DTPA-Pentetreotide (Octreoscan^®^). Octreoscan^®^ scintigraphy is a functional image that has a relevant role and high diagnostic precision in NETs of different origins. It is characterized by its specific binding to SSTRs 2 and 5 and, until recently, it represented the standard diagnostic method “gold standard” imaging [13,14] despite its limited resolution and the physiological uptake in some organs (liver, spleen, kidneys) that makes its interpretation challenging.

**Figure 3:**
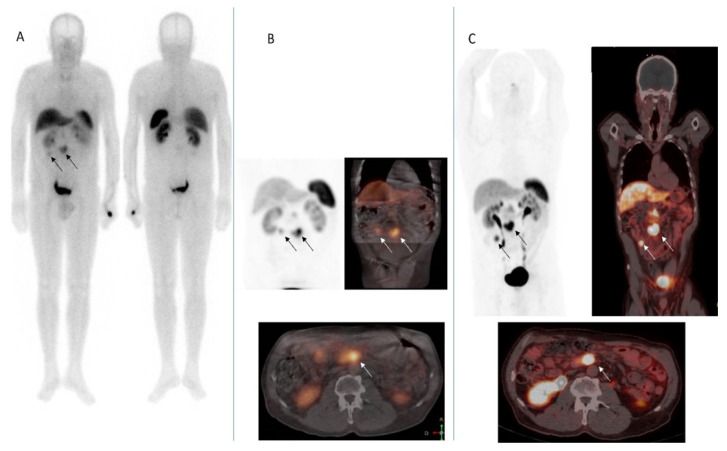
Comparison between somatostatin receptor functional images in a patient with midgut NET. (**A**). [99mTc] EDDA-HYNIC-Thr3-octreotide whole body scan in anterior and posterior projection. (**B**). [99mTc] EDDA-HYNIC-Thr3-octreotide SPECT/CT. Up to the left 3D reconstruction. Up to the right and down, SPECT/CT fusion in coronal and axial slices. (**C**). [68Ga]Ga-DOTA-TOC PET/CT. Up to the left maximum intensity projection. Up to the right and down PET/CT fusion in coronal and axial slices. The arrows point out the primary midgut NET and a mesenteric mass overexpressing somatostatin receptors.

Radiopharmaceuticals labeled with 99mTc were developed posteriorly, aiming to use gamma-emitting isotopes of higher image quality and easier handling. The [99mTc] EDDA-HYNIC-Thr3-octreotide (Tektrotyd^®^), represents an alternative to Octreoscan^®^ adding several advantages such as lower effective dose, easier preparation and speed in the acquisition of the study [15,16] (Figure 3).

The introduction of hybrid imaging systems (SPECT/CT) further contributed to improve the clinical validity of this technique [17]. The combination of scintigraphy with anatomical images (SPECT/CT) allows the correlation of functional and anatomical imaging, playing an important role in the localization of the primary NET and its metastases, as well as in the monitoring of treatment response. The use of SPECT/CT significantly improves the specificity and accuracy of isolated scintigraphy, since it reduces the rate of false positives and negatives, resulting in an improved image and achieving a more precise differential diagnosis of lesion [18,19].

Another step forward is positron emission tomography (PET), which uses positron-emitting radioisotopes (18F, 11C, 68Ga), a new generation of tracers with a shorter half-life and with less radiation for the patient. In addition, PET improves on conventional scintigraphy as it offers greater spatial resolution than previous techniques (3–6 mm lesions versus 10–15 mm), detects more lesions, and allows quantification of uptake in the same (standard uptake value, SUV), which is very useful in monitoring [14].

The recent introduction of novel somatostatin analogue (SSA) labelled with 68Ga has revolutionized the diagnostic approach to NENs. Different analogues have been developed [20,21] called 68Ga-DOTA-TOC, 68Ga-DOTA-TATE and 68Ga-DOTA-NOC. 68Ga-DOTA-NOC is the most useful and promising in NENs, due to its affinity for SSTR 2, 3, and 5, and its more favorable dosimetry. Currently, recommendations are made to replace 111In-pentetreotide SPECT/CT scan by 68Ga-DOTA-SSA PET/CT due to its higher sensitivity, diagnostic accuracy, and lower radiation (Table 1). However, the overall diagnostic accuracy of SSTR SPECT/CT and 68Ga-DOTA-SSA PET/CT is difficult to assess. This may vary depending on the location of the lesion, the degree of tumor differentiation and therefore the expression of SSTR, and the radiopharmaceutical used in PET/CT. All series show significant differences in sensitivity in favor of 68Ga-DOTA-SSA PET/CT, but no substantial differences in specificity have been observed. Regarding the impact on management, in four studies on 278 patients performed 68Ga-DOTA-SSA PET/CT in addition to previous 111In-DTPA-Pentetreotide SPECT/CT, an average of 39% of patients experienced changes in treatment strategy [22,23,24].

The use of 68Ga-DOTA-SSA PET/CT has several advantages, such as better spatial resolution, shorter duration of image acquisition and better availability since it is produced by a generator instead of cyclotron. Also, its ability to penetrate tissues is higher and in addition, 68Ga-peptide has a greater affinity for SSTR. Finally, SUV is an assessment of the intensity of SSTR overexpression and is useful for planning therapy and follow-up [25,26] (Figure 3).

The combination of the complementary 68Ga-DOTA-SSA PET/CT and 18F-FDG PET/CT as a single parameter, assess two different aspects of tumor biology: the expression of SSTR and glucose metabolism. The dual PET/CT radiotracer may be used for precise staging at initial diagnosis in those patients with intermediate tumor proliferative activity (especially for G2) and heterogeneous SSTR expression, and during the follow up, in addition to conventional imaging, at the time of restaging, progression after prolonged stable disease or in cases of discrepancy between radiological evaluation and clinical assessment [27,28].

## 4. Somatostatin Analogues as Treatment for NENs

The clinical use of native SST is limited due to its short half-life (1–3 min). Therefore, somatostatin analogues (SSA) have been developed with a longer half-life and affinity for specific SSTR subtypes, which define their clinical use.

At the end of the 1980s, the first SSA were developed, they present a similar molecular structure with a high affinity for SSTR2, moderate for SSTR5 and low for SSTR3 [29,30,31,32,33].

The first synthetic SSA was octreotide. There are two formulations available: one for short-acting and the other for prolonged-release, intramuscular administration (LAR). Recently, a new formulation for oral administration has been developed but is not yet approved for clinical purposes. Lanreotide was subsequently developed and has a longer half-life than octreotide. It is currently available in a sustained-release formulation for deep subcutaneous administration (Autogel). Long-acting formulations are usually administrated every 28 days. Both molecules show a good safety profile with similar and generally mild side effects, mainly due to the actions of SST at the gastrointestinal level. Side effects are dose-dependent and appear 24 h after administration, although they are frequently self-limited [29,30,31,32,33].

SSAs have shown both an antisecretory and antiproliferative effect on NENs, which have positioned this treatment as first line therapy on metastatic gastroenteropancreatic NENs.

SSAs due to their inhibitory effect on hormonal secretion may be useful for the symptomatic control of functioning NENs due to their antisecretory effect. Both octreotide and lanreotide offer similar results in terms of symptom control in patients with carcinoid syndrome, with a decrease in diarrhea in 75% and in flushing in 80% of cases reflected in different clinical studies. A recent meta-analysis reveals that octreotide achieves an improvement in symptoms in 65–72% of patients and a biochemical response in 45–46%. Notably, lanreotide showed very similar effects [34].

However, a decrease in response has been observed with prolonged treatment, with a mean duration with respect to hormonal hypersecretion of 12 months. This is attributed to a possible tumor progression, tachyphylaxis or treatment resistance. The possible physiopathological mechanisms, which explain tachyphylaxis is a possible down-regulation of SSTR expression on the tumor surface and/or the production of antibodies against SSAs. Different strategies have been investigated to improve efficacy after decreased response such as change one SSA to the other, dose escalation or decreasing the injection interval to 21 days [35].

SSAs exert an antiproliferative effect by different mechanisms, so they can be useful for tumor control both in functioning and non-functioning NENs. These effects have been demonstrated mainly on gastroenteropancreatic NENs. Since the beginning of their commercialization, multiple phase II studies and case series have reflected its antiproliferative effect in terms of disease stabilization, suggesting a beneficial effect in controlling disease progression [36,37,38,39,40,41]. However, it was not until randomized phase III clinical trials were developed that this effect was demonstrated and that these treatments were positioned on clinical guidelines as a first-line option.

The most important randomized clinical trials were PROMID and CLARINET, their characteristics are mentioned in Table 2. The PROMID trial was a placebo-controlled, prospective, randomized study in patients with metastatic neuroendocrine midgut tumors. Eighty five patients with well-differentiated NETs were randomized to either receive octreotide or placebo. The primary endpoint was time to tumor progression. Octreotide was associated with a significant longer time to tumor progression, 14.3 months, compared to the placebo (6.0 months). After 6-month follow-up, significantly lower tumor progression rates were observed within the octreotide arm (37% versus 66%). Patients with resected primary tumor and a lower tumor burden displayed a more favorable outcome [42]. The CLARINET was a controlled study of lanreotide antiproliferative response in neuroendocrine tumors, which included advanced G1/G2 differentiated, nonfunctioning, somatostatin receptor–positive NETs and documented disease progression status. The primary endpoint was progression-free survival. The lanreotide arm was linked to significantly prolonged PFS compared to the placebo, with estimated rates of progression-free survival at 24 months of 65.1% in the lanreotide group and 33.0% in the placebo group [43]. 

This antiproliferative effect has also been explored in other primary locations, such as lung NENs. The SPINET trial is a Phase III, prospective, multi-center, randomized, double-blind, study evaluating the efficacy and safety of LAN plus best supportive care versus placebo plus best supportive care for the treatment of well-differentiated, metastatic and/or unresectable, typical or atypical lung NETs. Last year the recruitment was finished and results are pending [44].

Meanwhile, an escalation of SSA dosage has been suggested to further improve antiproliferative effects. However, up to the present date, only a small number of studies are available, which in turn have shown contradictory results. A meta-analysis published in 2017 analyzed 18 trials using doses of more than 30 mg octreotide or 120 mg lanreotide over 28 days [45]. Disease control rates ranged from 30 to 100%, however, response rates were modest (0–14%). Rates of biochemical improvement (27–100%) and symptom improvement (23–100%) ranged widely depending on the population studied and the definition of response. These results opened the need for a large, prospective trial investigating the role of escalated-dose somatostatin analogues in the treatment of metastatic GEP NENs.

In September 2020, the results of the CLARINET FORTE study were communicated on ESMO. This study investigated the efficacy and safety of increasing the dose frequency of Somatuline^®^ Autogel^®^ (lanreotide) in patients with pancreatic or midgut NETs with progression within the last two years while on a standard lanreotide regimen for ≥24 weeks. Investigators concluded that increasing the dose frequency of lanreotide from monthly to bi-monthly achieved a progression-free survival of 8.3 months in patients with progressive midgut neuroendocrine tumors (NETs) and 5.6 months in patients with progressive pancreatic NETs [46].

Pasireotide, a new SSA, has recently been developed with a higher affinity for SSTR1, SSTR3 and SSTR5 compared to octreotide and lanreotide analogues and a similar affinity for SSTR2. It is available in a short-acting formulation for subcutaneous administration and a prolonged-release formulation for intramuscular administration. However, so far, pasireotide has not proven superior to octreotide or lanreotide in antisecretory or antiproliferative therapy. Therefore, the use of pasireotide in patients with NENs is not currently approved, although it has been approved in other endocrine diseases such as acromegaly and Cushing disease. Moreover, it is associated with a worse safety profile due to the increased risk of hyperglycemia [47,48,49].

## 5. Peptide Receptor Radionuclide Therapy

The first radiopharmaceutical used in this field was 111In-Pentetreotide and its clinical efficacy was attributed to the effect of Auger and conversion electrons. It was abandoned in Europe as a therapy option in favor of the more efficient β emitters 90Y and 177Lu. Peptide receptor radionuclide therapy (PRRT) on GEP NENs target SSTR2 binding actively to it. The radiopharmaceutical used in PRRT involves a triple structure: radioactive metal, whose radiation (β) allows the destruction of tumor cells; a chelating agent with a dual role, which binds the radiometal and allows it to bind to the carrier through a functionalized arm; and a targeting moiety (peptide), which is aimed at SSTR2 [50].

Compounds available include 90Y-DOTATOC (PRRT-Y), which were firstly developed, and [177Lu]Lu-DOTA-TATE (PRRT-Lu), which is the only PRRT approved for GEP NET patients in Europe and North America (Lutathera^®^). This section is not mandatory but can be added to the manuscript if the discussion is unusually long or complex.

**Figure 4:**
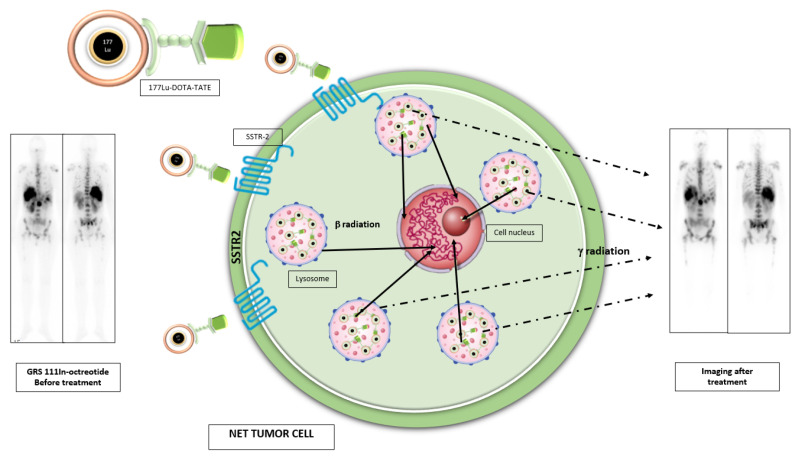
Schematic representation of PRRT-Lu treatment and whole body distribution scan 24 h post-PRRT treatment.

The molecular basis of therapy with [177Lu] Lu-DOTA-TATE is internalization and retention of radiolabeled somatostatin analogs in lysosomes of cells which express SSTR-2. After internalization within cells, β-irradiation produces the breakdown of intracellular DNA chains and cell death and it also produces low energy gamma radiation, which enables imaging. Consequently, it is essential for the efficacy of the therapy that both tumors and their metastases express SSTR, and show intense uptake on somatostatin receptor imaging (Figure 4) [51,52].

The development of analogue [DOTA]0-Tyr3-octreotate or DOTATATE labeled 177Lu was performed in the year 2000 [53]. [177Lu]Lu-DOTA-TATE has been investigated in several clinical phase I and II studies, case-control and cohort studies, several large well-designed cohort studies and a published meta-analysis undertaken in 2015, which describe that treatment with this radiopharmaceutical is an effective option for patients with inoperable or metastatic NENs achieving a more than acceptable disease control rate Table 3 [54,55,56,57,58,59,60,61,62,63,64,65,66,67,68,69,70,71,72,73].

However, it was not until NETTER-1, the first randomized phase 3 clinical trial on PRRT, that this treatment was approved. This randomized, phase 3 trial investigated PRRT-Lu every 8 weeks for 4 cycles plus octreotide 30 mg every 4 weeks versus high-dose octreotide LAR (60 mg every 4 weeks) in patients with advanced, SSTR imaging-positive, midgut NETs who progressed on standard-dose octreotide LAR [74]. PRRT-Lu produced markedly higher progression-free survival (estimated in 40 months) to alternative treatment with somatostatin analogues (SSA) (8.4 months). Recent data of OS of NETTER 1 have been communicated on American Society Congress of Medical Oncology (ASCO) 2021, median OS in the control arm was 36.3 months and 40 months in the PRRT-Lu arm [HR 0.84 (95% CI: 0.60, 1.17) with *p* = 0.30]. The difference of 11.7 months on OS was not statistically significant, probably due to a high rate (36%) of cross-over of patients in the control arm to PRRT after progression [75].

Based on the results of the NETTER-1 study, treatment with [177Lu]Lu-DOTA-TATE was approved by the Food and Drug Administration, by European Medicines Agency in 2017–2018 and has rapidly been incorporated on the most relevant clinical guidelines [76,77,78,79,80,81,82]. Guidelines consider the most important inclusion criteria: inoperable/metastatic well-differentiated (G1/G2) NET, sufficient tumor uptake on the diagnostic SSTR functional images, creatinine clearance >50 mL/min, sufficient bone marrow reserves, Karnofsky performance status >50 and expected survival >3 months. The most recent guidelines, those of ESMO published in 2020 [82], agree that PRRT-Lu can be recommended in patients with midgut metastatic NETs with disease progression on SSAs who fulfil the general requirements. PRRT-Lu can also be considered at further therapy lines and in NETs from other sites than the midgut. Results from randomized clinical trials in pancreatic NETs are lacking; therefore, in pancreatic NETs, molecular targeted agents such as everolimus or sunitinib, and systemic chemotherapy may be prior treatment choices, and PRRT-Lu should be considered after failure of these therapies.

## 6. Future Use of Alfa-Emmitters Binded to Somatostatin Antagonists

The use of alpha emitting radionuclides in PRRT could potentially increase the level of tumoral cell death by increasing the number of DNA double-strand breaks. Alpha radiations can easily be shielded, however, they transmit lots of energy with a great penetrating power, and in this way they could be used for treatment. These radionuclides include 212Lead-212 (212Pb), 213Bismuth (213Bi) and 225Actinium (225Ac). Promising results with 213Bi have been published in which enduring responses were observed in all treated patients with moderate kidney toxicity and with even less acute hematotoxicity than with the preceding beta therapies. Recently, a phase 1 study has been completed and results are pending. [^212^Pb]Pb-DOTAM-TATE has been tested. The primary objective was to assess the dose limiting toxicity using ascending doses of this radionuclide. The secondary objectives were to determine preliminary efficacy as well as the pharmacokinetic properties [83,84,85].

## 7. Future of the Use of Somatostatin Receptors as Targets on Neuroendocrine Tumors: Somatostatin Antagonists

An important development in the field of SSTR targeting is the recent introduction of SSTR antagonists. SSTR antagonists seem to recognize more binding sites on receptors and show better tumor visualization than agonists and more favorable pharmacokinetics despite a poor internalization rate [86,87].

Several SSTR antagonists have been studied since 1996, in which Bass was developed first [88]. Of all the somatostatin antagonists developed so far, the analogue JR11 performed the best in preclinical settings. JR11 has been selected both for clinical development as a PET imaging agent labeled with 68Ga using the chelator NODAGA (68Ga-OPS202 or 68Ga-NODAGA-JR11) and as a therapeutic agent labeled with 177Lu using the chelator DOTA (177Lu-OPS201 or 177Lu-DOTA-JR11) [89].

The first pilot study with antagonists in humans was performed with [177Lu] Lu-DOTA-JR11, which showed promising results with a 1.7–10.6 times higher tumor dose compared to the agonist [177Lu] Lu-DOTA-TATE [90]. It seems that antagonists generate higher tumor doses and larger numbers of DNA double strand breaks than agonists, resulting in better treatment efficacy [91,92]. Indeed, in a first human study with 4 patients who had metastastatic neuroendocrine neoplasia (grades G1–G3) antagonist 177Lu-DOTA-JR11 was more effective than 177Lu-DOTATATE. With antagonist 177Lu-DOTA-JR11, higher tumor doses, higher tumor uptake, higher tumor-to-kidney dose ratio, and higher metabolic stability were observed [93].

This novel agent has been explored to see if the [177Lu] Lu-DOTA-JR11 drug is safe to perform scans and treat NETs in an interventional study designed in three steps, NCT02609737, in which experimental imaging with [68Ga] Ga-DOTA-JR11 and experimental treatment with [177Lu]Lu-DOTA-JR11 are going to be explored. The primary outcome is overall response rate according to RECIST 1.1 and median PFS and OS. This study has been completed and results are pending [93].

The main concern over the use of these somatostatin antagonists is an increase in the uptake in bone marrow or kidney, which would increase their toxicity [92]. Future studies are aimed at reducing toxicity by reducing activity and longer treatment intervals which are expected to drastically reduce hematologic toxicity while preserving activity.

## 8. Conclusions

Due to the overexpression of SSTR-2 in NENs, somatostatin and somatostatin receptors have become the main targets to develop diagnostic and therapeutic strategies in these patients. In this way, SSA and PRRT have become first line and further line therapies approved by scientific societies and have been widely used in clinical practice. The possibility of using SSTR as well for imaging has provided extensive information of NEN metabolism and guidance for the use of the treatments mentioned before.

However, until recently, it was thought that internalization of the radiotracer was mandatory for SSTR imaging and therapy and recent investigations have described that radiolabeled SSTR antagonists to have the potential to optimize both diagnostic and treatment strategies. Therefore, although the efficacy of the use of SSTR agonists as a therapeutic target for imaging and treatment is unquestionable, a new future opens before us with the use of SSTR antagonists.

## Figures and Tables

**Table 1:** Overall sensitivity and specificity comparing 111In-DTPA-Pentetreotide SPECT/CT and 68Ga-DOTA-SSA PET/CT.

SSTR Imaging	Sensitivity	Specificity
68Ga-DOTA-SSA PET/CT	93% (70–100%) [22]	96% (67–100%)
111In-DTPA-Pentetreotide SPECT/CT	72% (58–75%) [23]	93% (77–99%)

**Table 2:** Comparison between PROMID and CLARINET studies. PPI: proton pump inhibitors.

Characteristics	Promid	Clarinet
Number of patients	85	204
Localization	Midgut	Midgut, foregut, pancreas, primary unknown
Grade	1 (Ki-67 ≤ 2%)	1, 2 (ki67 < 10%)
Funcionality	Functioning (38.8% carcinoid síndrome) Non functioning	Non functioning (Except gastrinomas well-controlled with PPI)
Liver burden	≤25%: 67.1% <10%: 67.2%	≤25%: 66% <10%: 51.9%
SSTR expression	Positive/negative	Positive (Krenning score 2–4)
Treatment	Octreotide LAR 30 mg/28 days versus Placebo	Lanreotide 120 mg/28 days versus Placebo
Primary objective	Time to progression (months)	Progression free survival (months)
Results	Stable disease: 66.7% vs. 37.2% Time to progression: 14.3 m vs. 6 m	Progression free survival 32.8 m vs. 18 m

**Table 3:** Results comparing disease response and control rate of PRRT in NETs.

Study	Study Design	Dose (GBq)	Number of Cycles	Nº Patients	Disease Response Rate (%)	Disease Control Rate (%)
Sward (2010) [64]	Retrospective	8	4–5	16	RECIST 37.5	RECIST 87.5
Van Vliet (2013) [65]	Retrospective	3.7–7.4	4	257	RECIST 27.6 SWOG 25.3	RECIST 76.2 SWOG 74
Ezziddin et al. (2014) [66]	Retrospective	8	4	68	RECIST 57.4 SWOG 60.3	RECIST 85.3 SWOG 85.3
Sabe et al. (2015) [59]	Retrospective	7.9	4	61	RECIST 16.4 SWOG 13	RECIST 90 SWOG 91.2
Bodei et al. (2016) [67]	Retrospective	3.7–6.5	4	54	RECIST 18.5	RECIST 72.2
Soydal et al. (2016) [68]	Retrospective	7.4	4–8	29	RECIST 27.6	RECIST 89.6
Hamditibar et al. (2017) [69]	Retrospective	7.4	1–6	28	RECIST 28.6	RECIST 114.3
de Prette et al. (2017) [70]	Retrospective	5.9–15.9	4	23	RECIST 8.7 SWOG 8.7	RECIST 73.9 SWOG 73.9
Khalshetty et al. (2019) [72]	Retrospective	5.55	4	46	RECIST 43.5	RECIST 80.4
Sansovini et al. (2013) [57]	Prospective	3.7–5.5	5	52	SWOG 28.8	SWOG 80.7
Bodei et al. (2011) [56]	Phase I/II	3.7–7.4	4–6	51	RECIST 29.4	RECIST 82.3
Delpassand et al. (2014) [60]	Phase II	7.4	1–4	32	RECIST 28.1	RECIST 71.9
Paganelli et al. (2014) [58]	Phase II	3.7–5.5	5	43	SWOG 7	SWOG 83.7
del Prete et al. (2018) [71]	Phase II	7.4	4	11	RECIST 18.2 SWOG 9	RECIST 81.8 SWOG 81.8
Strosberg et al. (2016) [74]	Phase III	7.4	4	101	RECIST 17.8	RECIST 17.8

## Data Availability

Not applicable.
